# Optimized Cylindrical Diffuser Powers for Interstitial PDT Breast Cancer Treatment Planning: A Simulation Study

**DOI:** 10.1155/2020/2061509

**Published:** 2020-03-23

**Authors:** Fatimah S. Ismael, Hani Amasha, Wesam Bachir

**Affiliations:** ^1^Biomedical Photonics Laboratory, Department of Laser Physics and Technology, Higher Institute for Laser Research and Applications, Damascus University, Syria; ^2^Biomedical Engineering Department. Faculty of Mechanical and Electrical Engineering, Damascus University, Syria; ^3^Faculty of Informatics and Computer Engineering, Syrian Private University, Damascus, Syria; ^4^Faculty of Informatics Engineering, Al-Sham Private University, Al-Baramkeh, Damascus, Syria

## Abstract

**Purpose:**

It is well known that interstitial photodynamic therapy (iPDT) of large tumors requires effective planning to ensure efficient delivery of therapeutic dose to the target tumors. This should be achieved in parallel with minimal damage to the nearby intact tissues. To that end, clinical iPDT can be attained using cylindrical diffusing optical fibers (CDFs) as light sources. In this work, we optimize output CDF powers in order to deliver a prescribed light dose to a spherical volume such as a tumor node.

**Methods:**

Four CDFs are placed vertically inside the tumor node. The fluence rate is calculated using the diffusion equation. Therapeutic target dose is (20-50) J·cm^−2^. The optical properties (*μ*_a_ = 0.085 cm^−1^, *μ*_s_′ = 16 cm^−1^) of a breast tumor and the treatment time of 150 sec are used to calculate the fluence rate.

**Results:**

For four CDFs, the therapeutic target dose (20-50) J·cm^−2^ is delivered to more than 90%. This is the ratio of the total points that receive the target dose in proportion to the total points in the volume of the node of 3 cm in diameter, whereas, in larger nodes, the ratio is decreased to approximately 67%. Five CDFs are required to improve this ratio by more than 10%.

**Conclusion:**

Optimizing delivered powers enables the distribution of the therapeutic dose uniformly in the medium. In addition, this simulation study represents an essential part of a development dosimetry system for measuring and controlling the optical dose in the breast tumors.

## 1. Introduction

Photodynamic therapy (PDT), as a minimally invasive technique, is a widely and clinically accepted method for treating a wide range of cancers, such as brain and prostate, to name but a few.

It comprises a light source and a photosensitizer in order to produce toxic singlet oxygen which, in turn, leads to cancerous tissue destruction [1].

Practically, optical fibers in iPDT can be inserted inside bulky tumors to ensure efficient illumination of the whole volume of tumors. However, the success of this modality requires improved understanding of the light dosimetry together with planned arrangement of optical fibers inside tumors.

Moreover, owing to the undesired side effects which resulted from conventional techniques used for treating cancers such as X-ray based instrumentation and iPDT is commonly recognized as a viable alternative for treatment of prostate cancer [2]. Thus, intensive research by several groups has focused on treatment planning and a number of algorithms were developed mainly for prostate cancer as a result. The Cimmino algorithm has been commonly used for prostate iPDT and proved to be effective when applied to other cancers [2, 3]. Also, Altschuler et al. and Davidson et al. demonstrated a combination of ultrasound imaging and a diffusion approximation for light propagation [2, 4] for their iPDT treatment planning system of prostate tumors.

Also, Baran and Foster demonstrated treatment planning software based on a graphics processing unit- (GPU-) enhanced Monte Carlo (MC) simulation framework for light propagation through complex 3D tissue volumes in the head and neck [5]. Oakley et al. concluded that image-based treatment planning can be used to compute the delivered light dose during interstitial photodynamic therapy (I-PDT) of locally advanced head and neck squamous cell carcinoma (LA-HNSCC). In their work, computed tomography (CT) was used to image the markers and phantoms. A finite element method was utilized to compute the light DVHs [6]. Jäger et al. reported a technique of iPDT for advanced head and neck tumors involving MR imaging-guided fiber placement. In their study, the extent of tissue damage corresponded to a radius of 9–11 mm. The needles and laser fibers were pulled back in 1 cm increments. Study results are encouraging and show that substantial necrosis of solid tumors can be achieved with imaging-guided light delivery by using CT or sonographic guidance and recurrent prostate cancer after radiation therapy by using transrectal sonographic or MR guidance [7].

Recently, photodynamic therapy has been investigated for breast cancer by several research groups with the aim of replacing the commonly used surgery that is widely practiced for removing tumor tissues, including breast-conserving surgery or complete removal of breast [8]. In addition, radiotherapy involves exposure to ionizing rays to destroy cancer cells, which is largely accompanied by unwanted short-term side effects such as swelling, heaviness in the breast, and even fatigue.

Dos Santos et al. included the applicability of PDT to the breast for the ablation of a broad range of solid tumors. The first clinical application of PDT for breast cancer treatment was to treat skin metastasis recurrence in the chest wall. The protocol tested was using Photofrin and showed benefits in fifty percentage of the patients [9, 10].

In addition, several groups developed spectroscopic techniques for photodynamic therapy dosimetry exactly for prostate treatment such as Johansson. Also, Ong et al. developed a 4-channel PDT dose dosimeter which was used during Photofrin-mediated pleural PDT [11, 12]. Owing to the fact that optimization algorithms could be effective to develop the techniques for iPDT breast cancer treatment, in this study, we introduce a new algorithm dedicated for optimal PDT treatment aimed at optimizing the delivered power to diffusing fibers and their lengths, thus optimizing the treatment planning for breast cancer photodynamic treatment.

## 2. Material and Methods

### 2.1. Cylindrical Diffusing Fibers

Recently, computer simulation works suggested that cylindrical diffusing fibers (CDFs) are more effective than other fibers in delivering the therapeutic light in iPDT especially for bulky tumors [2, 3, 5, 13]. The diffusers used in this study are cylindrical diffusing fibers made at Medlight S.A, Switzerland-Model RD-ML. Treatment fiber specifications are as follows: Transmission is defined in comparison with a 5 meter/600 microns/NA 0.37 silica bare fiber (630-760 nm), core diameter 400 *μ*m, maximum (CW) power density (in air) (0.5 W/cm), and absolute maximum input power 2.0 W (CW). Treatment regions include the breast, prostate, brain, heart, lung, and diaphragm. The diffuser length is 7 cm or less [14].

### 2.2. Tissue Model

**Figure 1:**
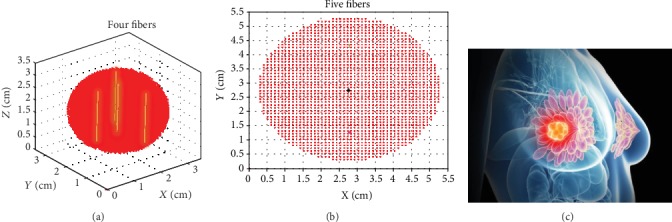
(a) 3D visualization of a tumor node. Node diameter is 3 cm. First CDF is placed vertically along the central diameter; three CDFs are placed vertically at the gravity center of 120° circular sectors. (b) Section of a central slice in the node. Node diameter is 5 cm. First CDF is placed vertically along the central diameter; four CDFs are placed vertically at the gravity center of 90° circular sectors. (c) The tumor node in the breast.

Heidari in his work revealed that breast tumors are not necessarily spherical in shape [15]. This study concerns the tumor of the lymph nodes, especially from stage AII where the lymph node is of a diameter ranging between 2 and 5 cm and has not been transmitted to the nearby nodes according to Ref. [16].  Figure 1 shows the hypothetical tumor node shape that was used in our simulation study. The node is divided into 0.1 mm^3^ fractional voxels. Four modeled cylindrical diffusers were embedded within the node. The first diffuser is placed along the central diameter, while the other three diffusers are placed at the gravity center of 120° angular sectors.

### 2.3. Diffusion Equation

The fluence rate generated by light sources can be estimated using analytical models of light propagation. These models are originally developed from the Boltzmann transport equation [17–19].

The basic equation for light diffusion in an infinite medium can be used to find the fluence rate at each point of the grid, in which the reducing scattering factor is much greater than the absorption factor. Optical properties of the medium need to be known. However, there is not much information available regarding the optical properties of different stages of breast tumor, but the two ranges given by Sandell et al. at 690 nm for the breast tumor are *μ*_*a*_ = [0.070–0.10] cm^−1^ and *μ*_*s*_′ [14.7–17.3] cm^−1^ [20] and the mean values of these ranges are *μ*_*a*_ = 0.085 cm^−1^ and *μ*_*s*_′ = 16 cm^−1^, respectively. These values are considered the values of the optical properties of the medium. In practice, the algorithm can be adjusted to any turbid medium with known optical properties.

The use of the Kernel method in order to divide the volume into parts, each with different optical properties, has been stated by different works, as stated by Li and Zhu [21]. An alternative way, in a heterogeneous medium, the optical properties are measured at several points, and then, the mean value is taken. This mean value may well represent the optical properties of the entire medium [22]. As for the current research, we consider that the algorithm can be developed so that we can apply it to other arbitrary shapes of the tumor by dividing the tumor into spherical volumes with different diameters and optical properties, as follows: The algorithm can be applied to each volume separately, where the diameter of each individual volume ranges between 0.5 and 5 cm and its diameter can be increased by 0.5 cm increment. Also, the application of this algorithm is not limited to the breast, but it can also be applied to other volumes with known optical properties.

**Figure 2:**
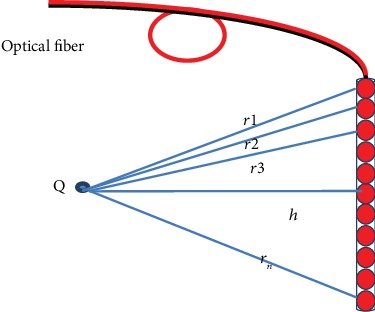
The optical fiber as a sum of several point light sources.

In Equation (1), cylindrical diffusor fiber was modeled as a series of point sources, to estimate the fluence rate emitted by a light source of a length *l* in homogeneous media. Equation (1) discretizes the diffusing part of the optical fiber as a sum of several point light sources, as shown in Figure 2 [2, 20, 23, 24].

(1) $$\begin{array}{c} \phi \left(r\right)=\frac{3.s.l.\mu_{s}^{\prime }}{4.\pi }.\frac{1}{N-1}.\overset{N}{\underset{i=1}{\sum }}\frac{e^{-\mu_{eff}.r_{i}}}{r_{i}}, \end{array}$$

where *ϕ*(*r*) is the fluence rate (mW/cm^2^); the quantity $\mu_{eff}=\sqrt{\;\left(3.\mu_{a}.\left(\mu_{a}+\mu_{s}^{\prime }\right)\right)\;}$ is the effective attenuation coefficient in tissues. *s* is the light power released per unit time per unit length (mW/cm). The differential *∆x* = *l*/(*N* − 1) is the length of the elemental (discretized) source segment. The odd integer *N* is the number of points used in the summation over the source, with one point always placed in the middle of the CDF. The distance between the *i*th point of the linear light source and the observing point is $r_{i}=\sqrt{x_{i}^{2}+h^{2}}$, where *x*_*i*_ = (*i* − 1 − (*N* − 1)/2). *∆x* is the cylindrical coordinate along the fiber from the center of the linear source and *h* is the distance perpendicular to the fiber axis. In Equation (1), the numerical value of the summation should be independent of *N* (or *∆x*) if *N* is large enough. Accurate results of the summation can be obtained if *∆x* = 0.05 cm. In this study, in all our calculations, *N* = 101 was used [2].

For simplicity, we use the light fluence (J·cm^−2^) (fluence rate × exposure time) for the PDT dose throughout the paper. The illumination time, through this study is 150 sec (this period is in the typical range of duration used in PDT) [6, 25]. Final light dose at each point is the summing of light doses that are received from all diffusers. To make this study closer to reality, threshold light dose is assumed to be 20-50 J·cm^−2^ [26] with optical properties of breast cancer at 690 nm [20]. The proposed algorithm can be a first step towards subsequent algorithms for possible future works that include states of tissue heterogeneity and the shape of edges as well. The algorithm is devised to optimize the delivered power and fiber length. Cimmino's method was suggested in which the rows can be accessed simultaneously and the next iteration vector is computed as the average of all the projections of the previous iteration vector [27]. In this work, the Cimmino algorithm for segmental anatomy was used for breast tumor as applied by previous works about prostate [2].

### 2.4. Time of Illumination

The assumed time to provide the optical dose is 150 sec continuous mode. It was reported in the reference of Tsutsui et al. that the tissue of the rat colon tumor was provided with the therapeutic dose during a continuous lighting period of 150 sec, but Foster and Baran revealed that the ability to choose the appropriate number of fibers is more important than determining the time factor [5]. In other studies, in Oakley et al., the time system was applied at 50:50:6000 sec, where the input energy per fiber is 400 mW/cm at 630 nm; the optical properties were *μ*_*a*_ = 0.2 cm^−1^ and *μ*_*s*_ = 27.77 cm^−1^, and the nonisotropic factor 0.82 [6]. However, Tsutsui et al. indicated the advantage of supplying the tissue with a low value of the therapeutic dose during a longer period of time. This was confirmed by [11].

## 3. Development of Treatment Planning Algorithm

### 3.1. Treatment Planning Algorithm

The treatment planning framework is based on the diffusion equation for light propagation through a homogenous spherical volume. This programming framework is used for simulating light distribution from individual treatment sources (CDFs) and for calculating the final dose after optimizing delivered powers. The development treatment planning algorithm consists of four major components: (1) determination of the optical properties, tumor node radius, distance between pixels, and the thickness of slice; (2) placement of the central diffuser and searching for optimal power; (3) placement of the surrounding fibers and optimizing their length and powers; and (4) calculation of fluence doses in every point and the ratios for every slice and for the node in total, displaying the diffusers within the node, the chosen required slice, and the histogram of the ratio of points which received the target dose of 20-50 J·cm^−2^. The diffusers were embedded vertically in the spherical volume (tumor node). Through this study, typical optical properties of breast cancer (*μ*_*a*_ = 0.085 cm^−1^, *μ*_*s*_′ = 16 cm^−1^) were used. Voxel dimensions were 0.1 × 0.1 × 0.1 cm. The thickness of every slice was 0.1 cm. The distributions of diffusers are uniform over the diffuser's length [14]. For optimizing the power of each diffuser power, power of central diffuser is independent of the surrounding diffusers. Powers of surrounding diffusers were equal. The length of the vertical central diffuser is equal to the diameter of the node. Surrounding diffusers were added at the same time at the centers of gravities of the angular sectors and their lengths were adjusted automatically to fit the volume of the node.

**Figure 3:**
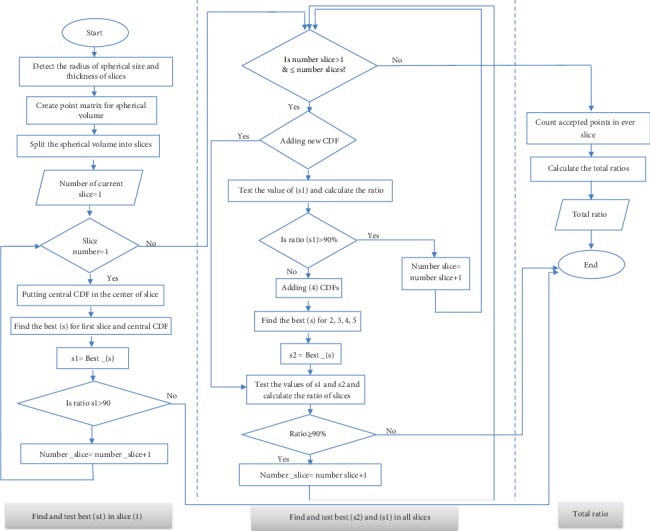
Flow chart of the developed algorithm.

**Figure 4:**
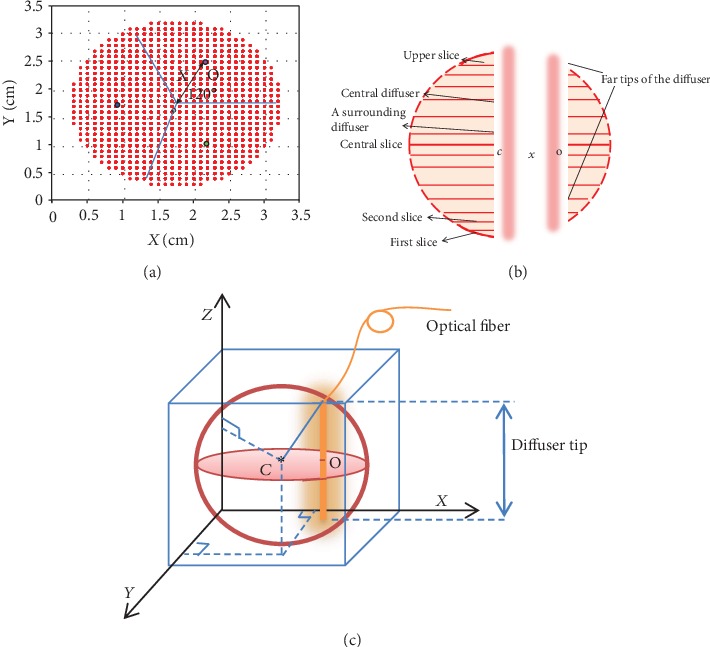
(a) Cross section of the central slice (*r* = radius of the spherical volume). The distance *x* is between the center *C* and gravity center of 120° circular sectors. (b) The index of the slices. (c) The geometry of the node.

**Figure 5:**
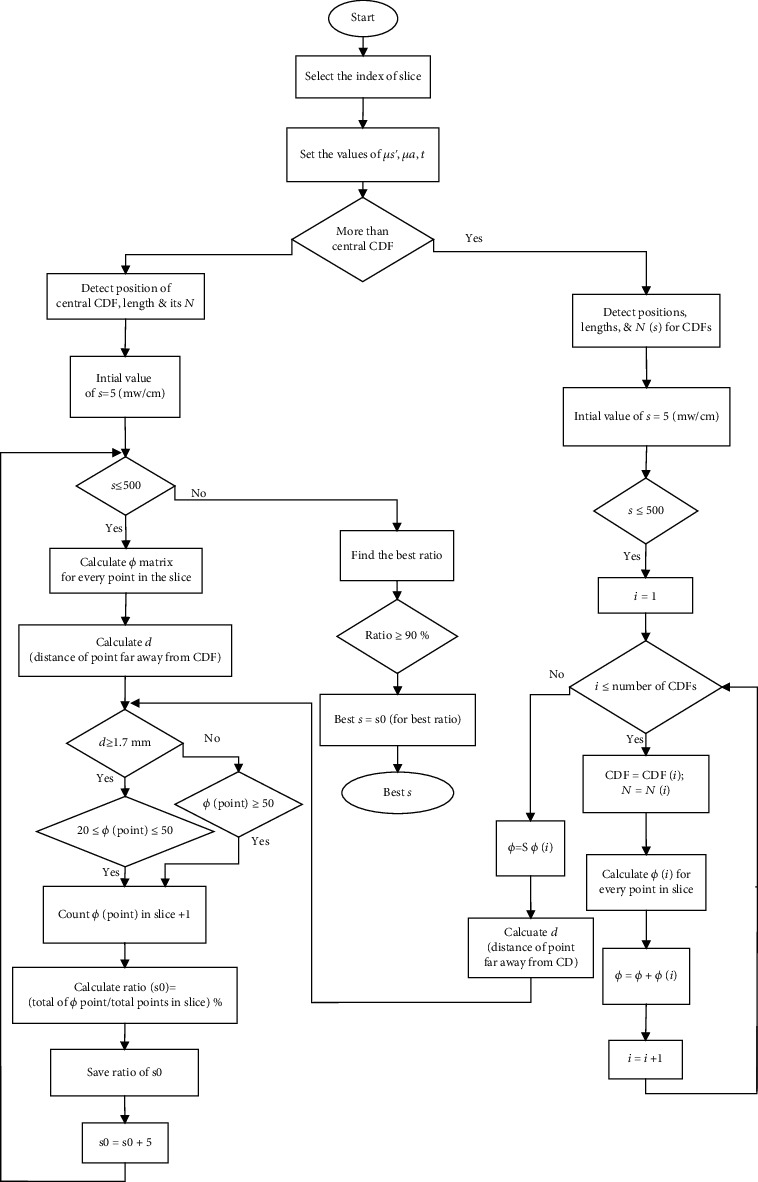
Flow chart of the calculations steps.

The following algorithm is a crucial part of the adjusted optoelectronic system incorporated in iPDT. The principle of the algorithm relies on the optimization of four individual cylindrical fiber powers to ensure the delivery of clinically accepted target light dose (20-50) J·cm^−2^ to—at least—90% [2, 5] of the spherical tumor volume. The power values of *s* (the light energy released per unit time per unit length (mW/cm)) varied between 5 and 500 mW·cm^−1^.  Figure 3 shows the flow chart of the developed algorithm. Main steps of the algorithm are labeled with numbers on the diagram and can be described as follows:

(1) The node is divided into (1 mm^3^) fractional voxels. Then, it is divided into a number of slices with a thickness of 1 mm for each
(2) The first fiber is placed along the central vertical diameter. Then, the code will search for the first desirable power to achieve the target light dose that covers 90% of the first slice located in the lower hemisphere, and then, the number of points fulfilling the condition of the target dose is multiplied by 2
(3) The resulting power from step (2), then, is tested on the adjacent upper slice to make sure that abovementioned target condition is fulfilled. This procedure is repeated for all slices until the condition breaks down
(4) Once the condition is not met, three additional fibers will be added at gravity centers of 120° circular sectors of the central slice (Figure 4(a)). The gravity center of the circular sector is calculated from Equation (2). Figure 4(a) shows the locations of the diffusers.
  (2) $$\begin{array}{c} x=\frac{2rsin\left(a\right)}{3a}, \end{array}$$
where *x* is the distance between the gravity center and the center of the circle and *r* is the radius of the central slice. Angle *a* equals half of the sector. The length of the added three fibers then is adjusted to fit the volume of the sphere, that is, until the far tips of each fiber intersect with the surface of the sphere (Figure 4(b)). This placement producer is depicted in Figure 4(c).
This step is followed by finding three equal powers that contribute to the central fiber power in order to deliver a target dose to 90% of the central slice. This can ensure that the powers of the fibers all together is adequate for delivering the target dose to all remaining slices of the volume.
(5) The algorithm is ended if the ratio of points that received the target light dose is ≥90%. The final step is counting target doses of 20-50 J·cm^−2^ in each slice and then calculates the ratio.  Figure 5 explains all calculations through the algorithm

### 3.2. Calculation Steps

Figure 5 represents the flow chart of the calculations steps.

1. Set the optical properties, illumination time, and index of a slice to display it after light distribution
2. Determine the number of diffusers at the current slice
3. Detect the positions and lengths of each fiber
4. Calculate the fluence rate *ϕ* at every point in the volume
5. Find the best power *s* that reaches the requirement target dose and count the total points that received the target dose. Find the total fluence rates at every point in the volume by adding the individual fluence rates
6. Calculate *d* (distance of point far away from the CDF). The near-source fluence rate (nsF) is within the first 0.17 cm distance [28].
7. Check the fluence rates at points within the first 0.17 cm distance and add the fluence rate which is more than 50 J.cm^−2^ to the total points that reach the requirement target dose
8. Calculate the ratio for the best *s* powers
9. Finally, display the best powers s1 and s2 (where s1 is the optimal power for the central CDF and s2 is the optimal power for the surrounding CDFs), the ratio for each slice, the total ratio for the volume, the selected slice in addition to the tumor volume, and the CDFs inside it

## 4. Results

Tables 1 and 2 list the results of the algorithm for optimizing the delivered powers of four/five cylindrical fibers. The four/five fibers were placed as explained as shown in Figures 1(a) and 1(b).

**Figure 6:**
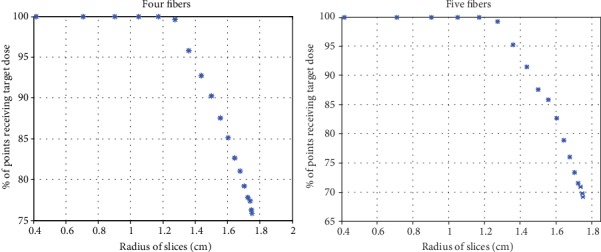
Comparison of optimized delivered powers between four and five fibers for node 7 with optical properties (*μ*_*a*_ = 0.085 cm^−1^, *μ*_*s*_′ = 16 cm^−1^).

Table 2 lists the results of the algorithm with four fibers. It is clear that target dose of 20-50 J·cm^−2^ could be reached to all points in nodes 1 and 2 using the central fiber only. So the three surrounding fibers are not added, while, for larger nodes 3, 4, 5, and 6 (Table 1) which have diameters 1.5, 2, 2.5, and 3 cm, respectively, target dose is delivered to more than 90% of all points. Therefore, the four fibers are sufficient. In node 7 (Table 1), since the diameter is 3.5 cm; target dose is reached to 85.5% of total points and target ratio 90% has not been achieved with the four fibers. Thus, a fifth fiber is added. Table 2 lists the results of the algorithm with five fibers. Four surrounding fibers are placed at the gravity centers of 90° angular sectors as shown in Figure 1(b). It should be noted that if a point that is located at a distance larger than 0.17 cm from the diffuser received an overdose (>50 J.cm^−2^), this point is dismissed. That explains why the ratio have decreased (after adding the fifth fiber) for all nodes which are smaller than node 7; in this case, adding the fifth fiber is not necessary. Comparison between four and five fibers can be seen in Figure 6. It can be seen that the ratio decreased after adding the fifth fiber. In Figure 6, every point represents two symmetrical slices. With four fibers, there are 18 slices that receive target dose while these slices are 16 with five fibers. This observation could be of great importance when the target dose at a specific number of slices of the volume is required for selective treatment inside a given volume.

**Figure 7:**
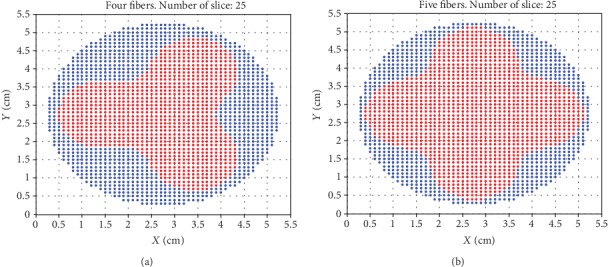
The central slice (number 25) in node 10. Red points received therapeutic target dose of 20-50 J·cm^−2^. The total number of points is 1976. (a) The total number of points that received the target dose is 1014. (b) The total number of points that received the target dose is 1304.

From Tables 1 and 2, one can note the improvement in the ratio after adding the fifth fiber for nodes 8, 9, and 10 since the diameters are 4, 4.5, and 5 cm, respectively.  Figure 7 shows the section of the central slice in node 10 in two cases: four fibers and five fibers.

**Figure 8:**
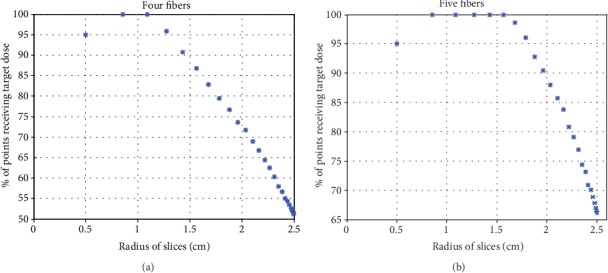
Comparison of ratios between four and five fibers for node 10. (a) With four fibers, five points represents ten slices which received 20-50 J·cm^−2^. (b) With five fibers, ten points represents twenty slices which received the same target dose.

For node 10, from the results in Table 2, it can be observed that the ratio is improved approximately at the rate 13% after adding the fifth fiber.  Figure 8 represents node 10 and shows the ratio of points that received target dose with four and five fibers. With four fibers, the numbers of slices that receive ratio ≥90% are 5, while they were 10 slices with five fibers. Also, for the wider central slice (radius is 2.5 cm), the ratio is approximately 50% with four fibers, but it is approximately 65% with five fibers. In this case, using five fibers is necessary to improve the ratio.

## 5. Discussion

**Figure 9:**
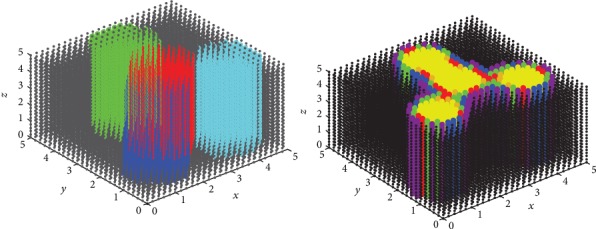
Illumination a homogeneous medium with four CDFs.

The accuracy of the calculated *in vivo* light fluence rate using the diffusion equation depends highly on the knowledge of tissue optical properties. Tissue optical properties can be very different from patient to patient and from tissue to tissue, and they are known to vary significantly due to disease progression as well. This study represents treatment planning for iPDT of a spherical volume in the human breast by modeling this volume and taking the updated optical properties as an input; then, the optimization is updated accordingly as a result. We adopted the optical properties of the breast tumor mentioned by Sandell et al. in the reference by taking the mean values of the ranges *μ*_*a*_ = [0.070–0.10] cm^−1^ and *μ*_*s*_′ [14.7–17.3] cm^−1^ at the 690 nm wavelength. Dos Santos et al. showed that the porphyrin (Verteporfin), a 2^nd^-generation PS, is suitable for this wavelength in the breast tumor. Lamberti et al. verified the suitability of this photosensitizer for breast tumor at 693 nm. Bhatti et al. also revealed conjugate selective cell death. The first step to develop the algorithm was to find out the effect of each variable in Equation (1); distance between observing point and the fiber axis *h*, diffuser length *l*, source power *s*, effective attenuation coefficient *μ*_eff_, and the reducing scattering coefficient *μ*_*s*_′. It was found that when placing four fibers as shown in Figure 9, in a homogeneous medium, the power of the middle fiber can be less effective than the power of the peripheral fibers, so that the dose is distributed homogeneously, but we have not reached the appropriate power values.

Although the therapeutic dose is related to the optical properties and wavelength, the dose 20-50 J·cm^−2^ has been determined, according to Filonenko et al., where doses were applied in the range between 20 and 30 J·cm^−2^ or dose of 50 J·cm^−2^. In this work, we assumed a homogenous tumor with certain values of optical properties for the treatment planning. However, in PDT, optical properties can be updated according to the measurements. We are in the process of developing an algorithm that has not yet ended, to infer the optical properties of a medium based on experimental measurements. As for optical properties, therapeutic dose, wavelength, and range of power are given, the algorithm will find the power values that meet the next fulfilling condition, that is; when the dose 20-50 J·cm^−2^ reaches 90% of the volume size and the fluence rate in all grid points will be calculated based on Equation (1). The fluence rate was considered in two regions: the near-source fluence rate (nsF) within the first 0.17 cm and the far-from-source fluence rate (fsF) beyond 0.17 cm. In the nsF region, diffusion theory seriously underestimates the fluence rate. In the fsF region, diffusion theory is more accurate and the explanation is given in Ref. [28].

The condition for the target dose was to deliver it to 100% of the prostate volume in Altschuler et al.'s study, but at a fixed power of the fibers at 150mw/cm^2^. Foster and Baran developed an optimizing algorithm for the locations of optical fibers depending on the Monte Carlo in the brain tumor to deliver the dose 90%, where the power value was chosen based on an fmincon algorithm. In their studies, 12 fibers were used to cover the prostate and 6 fibers to cover the size of a brain tumor 6.1 × 7.5 × 7 cm, respectively. In this study, however, four fibers were used to cover an increasing spherical volume of a diameter from 0.5 cm to 5 cm, with an increment of 0.5 cm. The maximum output power according to Medlight technical specifications was 500 mW/cm. As for tumor border, a condition can be set for the dose of edge points of the node that is equal or smaller than 20 J.cm^−2^, in order to avoid a thermal injury in the surrounding tissue.

Another point of consideration is the length of the fibers as they might not have the same length of fibers that are manufactured by Medlight. However, the findings of this work generate rough estimation of the required length. This approximation is proportional to the volume of interest. Time in the algorithm was a presumption. The results appeared in Figures 6–8 could be of great importance when the target dose at a specific number of slices of the volume is required for selective treatment inside a given volume. It should be noted that this research represents a hypothetical treatment plan for the treatment of precancerous tumors in PDT and the presented algorithm is an example of the ability of cylindrical radiators in particular. The presented study is in agreement with previous works. That is, in order to meet the desired treatment objectives, homogenous PDT dose needs to be cautiously determined in all parts of the tumor of interest.

## 6. Conclusion

This paper attempted to describe a novel and robust algorithm for optimizing the power and length of diffusing fibers used in iPDT.

The optimizing power values enable a uniform distribution of the therapeutic dose in a medium. This simulation study represents an important step towards the development of a dosimeter system for measuring and controlling the optical dose in the breast tumors. However, it can be applied to any other medium after updating the optical properties. The algorithm allows measuring the optical dose in a continuous treatment mode. Furthermore, future works may consider developing the algorithm to include heterogeneous medium and more complex arbitrary shape.

## Figures and Tables

**Table 1:** Four Fibers/Illumination Time = 150 sec/Target Dose [20 − 50] J · cm^−2^/*μ*_*a*_ = 0.085 cm^−1^, *μ*_**s**_′ = 16 cm^−1^.

Node number	Tumor diameter (cm)	Total slices in the node	Power of the central fiber reaches up to slice	Power of the central fiber (mW/cm)	Power of surrounding fibers (mW/cm)	Target dose reaches up to volume %	Length of the surrounding diffusers (cm)	The distance *x* (cm)
1	0.5	5	All	65	—	100	0.4170	—
2	1	10	All	70	—	99.3	0.8330	—
3	1.5	15	4	65	30	98.8	1.2520	0.4135
4	2	20	2	65	55	95.7	1.6701	0.5513
5	2.5	25	2	65	90	92.7	2.0802	0.6892
6	3	30	2	70	135	90.3	2.5029	0.8270
7	3.5	35	2	65	175	85.5	2.9200	0.9648
8	4	40	4	70	145	68	3.3372	1.027
9	4.5	45	2	75	195	67	3.7543	1.2405
10	5	50	2	80	235	63	4.1714	1.3783

**Table 2:** Five Fibers/Illumination Time = 150 sec/Target Dose [20 − 50] J · cm^−2^/*μ*_*a*_ = 0.085 cm^−1^, *μ*_s_′ = 16 cm^−1^.

Node number	Tumor diameter (cm)	Total slices in the node	Power of the central fiber reaches up to slice	Power of the central fiber (mW/cm)	Power of surrounding fibers (mW/cm)	Target dose reaches up to volume %	Length of the surrounding diffusers (cm)	The distance *x* (cm)
1	0.5	5	All	65	—	100	0.3999	—
2	1	10	All	70	—	100	0.7998	—
3	1.5	15	4	65	15	90	1.1998	0.4502
4	2	20	2	65	35	95	1.5997	0.6002
5	2.5	25	2	65	55	90	1.9996	0.7503
6	3	30	2	70	65	81	2.3995	0.9003
7	3.5	35	2	65	95	82	2.7994	1.0504
8	4	40	4	70	120	79	3.1994	1.2004
9	4.5	45	2	75	175	81	3.5993	1.3505
10	5	50	2	80	215	79	3.9992	1.5005

## Data Availability

The data used to support the findings of this study are available from the corresponding author upon request.
